# Evaluating a quality improvement intervention for schistosomiasis mass drug administration in Ghana using the RE-AIM framework

**DOI:** 10.1017/S0950268825100873

**Published:** 2025-12-23

**Authors:** Nicole Vernot-Jonas, Alfred Kwesi Manyeh

**Affiliations:** 1 Department of Global Health, Georgetown University, USA; 2 Institute of Health Research, University of Health and Allied Sciences, Ghana

**Keywords:** Schistosomiasis (bilharziasis), Mass drug administration, RE-AIM Framework, Volta Region, Ghana

## Abstract

Despite efforts by the Volta River Authority (VRA) to provide services for schistosomiasis control in communities along Ghana’s Volta Basin, high rates of transmission and re-infection persist in the region. To strengthen intervention effectiveness, the VRA partnered with the University of Health and Allied Sciences to conduct implementation research aimed at developing context-specific, evidence-based quality improvement strategies. This mixed-method study evaluates the reach, effectiveness, adoption, implementation, and maintenance of the VRA’s quality improvement intervention for their mass drug administration (MDA) for schistosomiasis. Baseline and endline surveys were analysed using STATA and qualitative data from in-depth interviews (IDIs) and focus group discussions (FGDs) were coded and analysed thematically using Taguette. Urogenital schistosomiasis prevalence decreased by 87.83% in Shai Osudoku, 88.98% in South Tongu, and 90.96% in Asuogyaman after the intervention. The findings revealed high training levels among district health management staff and community drug distributors, high health worker satisfaction with the training, and positive community reception of the intervention. However, praziquantel side effects and related opportunity costs may have posed a barrier to drug uptake. Moreover, re-infection remains a challenge, which could be attributed to high domestic and economic reliance on the Volta River.

## Introduction

### Background

Schistosomiasis, also known as bilharziasis, is a waterborne parasitic disease caused by blood flukes of the genus *Schistosoma* [1]. Three primary schistosomes infect humans: *Schistosoma mansoni*, *S. japonicum*, and *S. haematobium.* A neglected tropical disease (NTD), schistosomiasis primarily affects poor and rural communities with limited access to safe water and sanitation [2]. Individuals become infected when the aquatic form of *Schistosoma* (cercariae), released by snail intermediate hosts into fresh bodies of water, penetrate the skin upon contact [3, 4]. Once inside the body, cercariae mature into adult worms that inhabit either the mesenteric veins (*S. mansoni* and *S. japonicum*) causing intestinal schistosomiasis or the venous plexus of the bladder (*S. haematobium*) causing urogenital schistosomiasis [5]. The transmission cycle continues when humans infected with schistosomiasis urinate or defecate in or near bodies of water, passing *Schistosoma* eggs back into the environment [1]. Symptoms of schistosomiasis vary: intestinal schistosomiasis is associated with abdominal pain, diarrhoea, and bloody stool, while urogenital schistosomiasis is typically characterized by blood in the urine and can lead to bladder cancer, increased risk of human immunodeficiency virus (HIV), and infertility [2, 6].

Globally, 1.8 million disability-adjusted life years (DALYs) are attributable to schistosomiasis [7]. *Schistosoma mansoni* is found in Africa, parts of South America (Brazil, Venezuela, Suriname), the Caribbean, and the Arabian Peninsula; *S. japonicum* is found in China, the Philippines, and Indonesia [2, 8]; and *S. haematobium* is found in Africa and the Arabian Peninsula [8]. The World Health Organization’s (WHO) 2021–2030 road map for NTDs aims to eliminate schistosomiasis in endemic countries by 2030 [9]. Recommended control strategies include mass drug administration (MDA) of praziquantel as preventive chemotherapy, complemented by water, sanitation, and hygiene (WASH) interventions, vector control targeting freshwater snails, and health education campaigns [3].

Despite global efforts, the burden of schistosomiasis remains highly concentrated in Africa, which accounts for approximately 85% of cases worldwide [3, 7]. Within the continent, Ghana bears a particularly high burden of disease: both *S. mansoni* and *S. haematobium* are common, and the country has the third highest prevalence of schistosomiasis for sub-Saharan Africa at 23.3% [10]. Transmission is high in communities close to freshwater bodies where limited access to potable water leads to reliance on water bodies for domestic and economic activities such as fishing, agriculture, clothes washing, mat weaving, and swimming, all of which expose individuals to schistosomiasis if the river is infected [2]. In some communities, such as those found along the Volta Basin, prevalence of schistosomiasis can be as high as 78%. Studies have shown that the prevalence of schistosomiasis in the Volta Basin is elevated in part due to the construction of the Akosombo and Kpong dams by the Volta River Authority (VRA) that created the Volta Lake [11]. The new breeding site for the vectors of disease, increased fishing activity, and the relocation of several villages for construction of the dam contributed to a higher prevalence and transmission of disease [11]. To combat the high prevalence of disease, the Ghana Health Service launched an annual MDA and an educational campaign for young school kids in 2008, and the VRA Lakeside Health Unit also offers targeted health and educational interventions [10, 11]. While some progress has been made, control efforts have not achieved the intended impact, with high rates of transmission and re-infection persisting in the Volta Basin communities.

### Study aim

The main objective of this article is to use the Reach, Effectiveness, Adoption, Implementation, Maintenance (RE-AIM) framework to evaluate the impact of the VRA intervention, which consists of four strategies: (1) engagement and involvement of community leaders and other stakeholders, (2) social mobilization and sensitization, (3) training of drug distributors (CDDs) and community volunteers, and (4) improve drug distribution process. Table 1 offers details on the specific actions related to each strategy as well as the desired implementation outcomes and effects (Table 1). The intervention package, which includes an MDA, was implemented by the VRA in partnership with Ghana Health Service in 2024.

**Table 1.** Volta River Authority (VRA) intervention strategies for schistosomiasis control in three regions of Ghana (model adapted from [12])

Domain	Engagement and involvement of community leaders and other stakeholders	Social mobilization and sensitization	Training of drug distributors (CDDs) and community volunteers	Improve drug distribution process
Action(s)	A. Identify, engage, and involve stakeholders such as Chiefs/Queen mothers, Assembly members, religious and traditional leaders before, during, and after the mass drug administration (MDA) B. Focus key messages on cause mode of transmission, treatment and prevention of the disease, importance of the MDA, and how to identify the disease, what to do, and minimize adverse drug reactions	A. Develop context-specific multi-channel communication strategy to create awareness among community members (using community information centres, community radio discussions, and announcements, in churches, mosques, schools, markets, etc.) B. Meetings with other social groups/professional associations to explain relevance of the MDA C. Focus key messages on cause mode of transmission, treatment, and prevention of the disease, importance of the MDA, and how to identify the disease, what to do, and minimize adverse drug reactions	A. Develop training material and schedule for community volunteers and drug distributors B. Train community volunteers and drug distributors to: i. Have good knowledge about schistosomiasis (transmission, prevention, treatment) and to be able to explain the importance of the schistosomiasis MDA exercise to community ii. Be able to convince every qualified person in the endemic area to participate in the MDA exercise iii. Know the inclusion and criteria of the MDA iv. Know the possible adverse drug reactions and explain them to the community members v. What to do in case of adverse drug reactions vi. Possible skills of communication and interaction and understand the importance of being patient and tolerant with difficult community members C. The training should include pre- and post-assessment of schistosomiasis and MDA knowledge and roleplays. Strong enforcement of the MDA procedures, in particular, Directly Observed Treatment (DOT) policy during training and supervision	A. An adequate number of days should be dedicated to the distribution exercise (not less than 1 week) B. The distribution should reach all eligible persons at homes, institutions, market places, offices, churches, mosques, and all other places in the communities C. People with higher level of educational qualifications and a good knowledge of the MDA should be sent to institutions and offices to distribute the drug D. Strong enforcement of the DOT strategy should be ensured E. There should be enough time for mop-up F. The MDA should be conducted regularly based on schistosomiasis prevalence in the affected communities as recommended by WHO and GRS
Implementation outcome(s) and effect	- At least 15% increase in MDA coverage and reduction in the number of refusals (increased participant responsiveness) - Reduce schistosomiasis prevalence by 10%	- At least 15% increase in MDA coverage and reduction in the number of refusals (increased participant responsiveness) - Reduce schistosomiasis prevalence by 10%	- Increase the level of adherence to schistosomiasis MDA implementation procedures and participants’ responsiveness - Increase in knowledge by 15% (pre–post training assessment)	- At least 15% increase in MDA coverage and reduction in the number of refusals (increased participant responsiveness) - Reduce schistosomiasis prevalence by 10%

## Methods

### Study design

This implementation research employed a mixed methods study design. The study compared pre- and post-intervention quantitative data alongside post-intervention qualitative data to evaluate the reach, effectiveness, adoption, implementation, and potential for maintenance of the intervention.

### Study areas

The data were collected in nine communities in the Asuogyaman, South Tongu, and Shai Osudoku districts of Ghana located in the Eastern, Volta, and Greater Accra regions, which are schistosomiasis endemic regions along the Volta Basin. Table 2 details the specific communities and the urogenital schistosomiasis prevalence for each community, which were purposively selected based on a urogenital schistosomiasis prevalence of greater than 50% (Table 2). In these communities, the VRA Lakeside Health Unit offers health services, vector control, and epidemiological investigations. Its interventions include the destruction of vector breeding sites, construction of WASH infrastructure, health education campaigns, and MDA of praziquantel [11].

**Table 2.** Urogenital schistosomiasis prevalence by study site

Region	District	Community	Urogenital schistosomiasis prevalence (%)
Eastern	Asuogyaman	Adome	78.6
		Nyamben	67.9
		Korankye	64.6
Volta	South Tongu	Dzetrokope	74.1
		Avegagome	62.1
		Asidohui	46.3
Greater Accra	Shai Osudoku	Lubuse	66.3
		Avakpo	55.8
		Atrobinya	54.7

### Study population and sampling

The study population comprised implementers and beneficiaries of the VRA intervention in the selected districts and communities. NTD focal persons, chiefs and opinion leaders, community members, health workers, community drug distributors (CDDs), head teachers, school health education programme (SHEP) coordinators, district directors of health service, and VRA public health officers aged 18 and older participated in both the baseline and endline surveys used for the quantitative data and in the IDIs and FGDs used for the qualitative data. Children both in and out of school aged 10 to 17 years were also eligible for inclusion.

### Data collection

This study utilized quantitative and qualitative secondary data. Quantitative data consisted of pre- and post-intervention MDA coverage and schistosomiasis prevalence by district as well as surveys administered to health workers and community members by trained research assistants. District-level MDA coverage and prevalence data were obtained directly from district health authorities. Qualitative data included in-depth interviews (IDIs) and focus group discussions (FGDs), also conducted by research assistants.

Data collection tools such as the semi-structured IDI and FGD guides, as well as the structured community survey questionnaire, were pre-tested in communities with similar characteristics to the study sites and revised based on the results. Baseline data from the quantitative survey were collected in August–September 2023 and endline data (including the quantitative survey and the qualitative IDIs and FGDs) were collected in November–December 2024.

A team of 12 graduate research assistants from the University of Health and Allied Sciences was recruited and trained for 6 days on research ethics and field data collection procedures. Following the training period, research assistants contacted community representatives, including community health officers or assembly members, and explained the study objectives. These representatives helped identify participants for the quantitative survey and qualitative IDIs and FGDs. Research assistants obtained informed consent from all participants prior to data collection.

Interviews were conducted in English or local languages, depending on participant preference, and subsequently translated. All IDIs and FGDs were recorded using a digital voice recorder and transcribed for analysis.

### Data management and analysis

Qualitative content analysis was done using Taguette, an open-source qualitative analysis software. Transcripts were analysed using an inductive content analysis approach, whereby the researcher read through the transcripts to identify emerging themes and patterns, created a codebook based on initial findings, and re-read the transcripts to further refine the codes and sub-codes.

Baseline and endline quantitative data from the community and training surveys were transferred to an Excel sheet for data cleaning, then exported to STATA 17 statistical analysis software (StataCorp, 2017, Stata statistical software: release 17; College Station, TX, StataCorp LP, Texas, USA) for analysis. Analysis involved tabulations of frequencies and percentages. Pre- and post-intervention surveys comprised independent cross-sections. To determine whether changes in data from baseline to endline were statistically significant, chi-square analysis was conducted to yield a *p* value. A *p* < 0.05 was considered statistically significant.

### Measurements

RE-AIM (reach, effectiveness, adoption, implementation, maintenance) is a framework that can be used to implement and evaluate interventions using five contextual indicators of success beyond simply effectiveness [13]. The framework was chosen for this study because it offers a comprehensive guide for understanding the strengths, implementation challenges, and overall impact of the VRA intervention, including the MDA and MDA-related education and training strategies.

#### Reach

To determine the reach of the intervention within the target population, quantitative indicators were calculated based on data obtained directly from district health authorities. These indicators included the proportion of the district management team trained, the proportion of CDDs trained, and changes in MDA coverage following implementation of intervention strategies.

#### Effectiveness

To assess effectiveness at the prevalence level, the per cent change in urogenital schistosomiasis prevalence after the MDA was calculated. Both quantitative and qualitative indicators were used to assess the effectiveness of the intervention at the behavioural level among both community members who participated in the MDA and health workers who were trained to deliver the MDA intervention. Changes in knowledge, attitudes, and practices (KAPs) of community members regarding schistosomiasis prevention and treatment after the MDA intervention were assessed using survey responses. Comprehensive knowledge of schistosomiasis was defined as the ability to correctly identify the mode of schistosomiasis prevention, transmission, and at least one symptom of schistosomiasis. Qualitative data from the IDIs and FGDs were also analysed to understand community KAPs regarding schistosomiasis and the MDA intervention itself. Post-training survey data were analysed to understand health worker perceptions of the training component of the intervention.

#### Adoption

Adoption of the programme by community stakeholders was assessed by analysing qualitative data from the IDIs and FGDs to understand community member and health worker perceptions of the VRA intervention. Community members were asked to describe their attitudes towards the MDA intervention and any barriers to their participation in the MDA component.

#### Implementation

The level of implementation (treatment fidelity) was assessed using data from the community survey, IDIs, and FGDs to understand the actual strategies used for the VRA intervention, including the methods used for social mobilization and the source of community information. Adherence to drug delivery strategies was also assessed using endline survey data.

#### Maintenance

The sustainability of the intervention was assessed by exploring challenges encountered during implementation and strategies employed to address them as reported by community stakeholders in the IDIs and FGDs. Suggestions for improvement were thoroughly reviewed and factored into consideration for the maintenance of this intervention.

### Ethical considerations

This study received ethical approval from the Ghana Health Service Ethics Review Committee (GHS-ERC: 025106122) and the Research Ethics Committee of the University of Health and Allied Sciences (UHAS-REC A.11 [104] 21-22).

## Results

### Demographic characteristics of study participants

The baseline survey reached a total of 960 respondents and the endline survey reached 906 respondents. The endline survey comprised 481 (53.15%) females and 424 (46.85%) males, with the mean age being 37.8 and ranging from 10 to 92. The majority of respondents were Christian, and the most common occupation was business, followed by farming. Table 3 summarizes the socio-demographic characteristics of the endline survey respondents across the three districts.

**Table 3.** Socio-demographic characteristics of endline survey respondents by district

Characteristic	Number (%) of respondents			
	Shai Osudoku	South Tongu	Asuogyaman	Total
Number of respondents	302 (33.33)	299 (33.00)	305 (33.66)	906 (100)
Age				
18–29	127 (43.20)	92 (31.29)	112 (37.97)	331 (37.49)
30–44	93 (31.63)	80 (27.21)	95 (32.20)	268 (30.35)
45–53	34 (11.56)	42 (14.29)	33 (11.19)	109 (12.34)
54+	40 (13.61)	80 (27.21)	55 (18.64)	175 (19.82)
Sex				
Female	154 (50.99)	172 (57.72)	155 (50.82)	481 (53.15)
Male	148 (49.01)	126 (42.28)	150 (49.18)	424 (46.85)
Education				
None	72 (24.00)	84 (28.19)	97 (32.12)	253 (28.11)
Primary school	55 (18.33)	57 (19.13)	69 (22.85)	181 (20.11)
JSS/JHS/middle school	94 (31.33)	96 (31.21)	76 (25.17)	266 (29.56)
SSS/SHS/technical/vocational school	65 (21.67)	42 (14.09)	53 (17.55)	160 (17.78)
Tertiary	14 (4.67)	19 (6.38)	7 (2.32)	40 (4.44)
Religion				
Christian	266 (89.26)	235 (79.12)	265 (86.89)	766 (85.11)
Muslim	10 (3.36)	1 (0.34)	21 (6.89)	32 (3.56)
Traditionalist	14 (4.70)	39 (13.13)	9 (2.95)	62 (6.89)
Other	8 (2.68)	22 (7.41)	10 (3.28)	40 (4.44)
Marital status				
Single	126 (41.72)	101 (33.89)	104 (34.55)	331 (36.74)
Married	126 (41.72)	146 (48.99)	155 (51.50)	427 (47.39)
Living together	13 (4.30)	6 (2.01)	6 (1.99)	25 (2.77)
Divorced	4 (1.32)	7 (2.35)	7 (2.33)	18 (2.00)
Separated	10 (3.31)	5 (1.68)	3 (1.00)	18 (2.00)
Widower	23 (7.62)	33 (11.07)	26 (8.64)	82 (9.10)
Occupation				
Artisan	18 (5.96)	36 (12.08)	29 (9.51)	83 (9.17)
Business	77 (25.50)	82 (27.52)	70 (22.95)	229 (25.30)
Farmer	66 (21.85)	59 (19.80)	52 (17.05)	177 (19.56)
Fishing	19 (6.29)	22 (7.38)	53 (17.38)	94 (10.39)
Government job/civil servant	13 (4.30)	11 (3.69)	11 (3.61)	35 (3.87)
Labourer	21 (6.95)	7 (2.35)	12 (3.93)	40 (4.42)
Student	28 (9.27)	15 (5.03)	31 (10.16)	74 (8.18)
Unemployed	37 (12.25)	41 (13.76)	23 (7.54)	101 (11.16)
Other	23 (7.62)	25 (8.39)	24 (7.87)	72 (7.96)

A total of 30 IDIs and 13 FGDs conducted post-intervention were analysed. Three interviews were with VRA staff, 18 interviews were with health workers, and 9 interviews were with community members. FGDs were conducted by age and gender, with FGDs conducted separately for male adults, female adults, male adolescents, and female adolescents in each district.

### Assessing the reach of the MDA programme

For each of the three districts included in this study, four out of four district health management staff were trained at the regional level, representing 100%. High rates of training were achieved at the community level in two districts, with 83.3% of CDDs trained in Shai Osudoku and 94.4% CDDs trained in South Tongu. While a lower proportion (30%) of CDDs were trained in Asuogyaman, a higher total number of CDDs were trained in the district: 90 CDDs compared to 25 and 51 in Shai Osudoku and South Tongu, respectively. A total of 21458 (51.9%) eligible community members across the three districts received praziquantel in the MDA, with the highest coverage levels achieved in Asuogyaman at 84.5% coverage (Table 4). Coverage increased compared to prior MDAs in Shai Osudoku and Asuogyaman, but decreased in South Tongu.

*Usually, when we are doing it on our own, we may have 40 to 50 communities. But this one, we went way beyond 200 communities. In terms of the number of persons we treat, we usually treat about 5,000 people. But this one is way beyond 20,000 people. So in terms of coverage, because Ghana Health Services is huge, and they are present everywhere, that’s why we’re leveraging on their coverage. (VRA Staff, Asuogyaman)*

**Table 4.** Mass drug administration (MDA) coverage before and after Volta River Authority (VRA) intervention by district

District	Pre-intervention coverage (%)^a^	Post-intervention coverage (%)	Type of change (positive +, negative −)
Shai Osudoku	32.59	50.6	+
South Tongu	68.4	30.84	−
Asuogyaman	41.7	84.5	+

a School-based MDA coverage.

### Measuring effectiveness of the MDA programme

Schistosomiasis prevalence decreased by 87.83% in Shai Osudoku, 88.98% in South Tongu, and 90.96% in Asuogyaman after the MDA intervention, decreasing from over 35% prevalence in each district to under 10% (Table 5). VRA staff acknowledged the success of the MDA intervention in lowering prevalence rates, citing the power of partnerships and evidence-based strategies.

*Following the MDA at the evaluation revealed that the prevalence rate had plummeted to 4.78%, a powerful testament to what can be achieved through dedicated partnerships, innovation, and evidence-based interventions. So you can see the benefits or the impacts of the collaboration. (VRA Staff, Asuogyaman)*

**Table 5.** Prevalence before and after mass drug administration (MDA) by district

District	Baseline prevalence	Post-MDA prevalence	Type of change (positive +, negative −)	Magnitude of change %
Shai Osudoku	39.84	4.85	**−**	87.83
South Tongu	37.76	4.16	**−**	88.98
Asuogyaman	60.53	5.47	**−**	90.96

The majority of survey respondents had correct knowledge of how schistosomiasis is acquired both before and after the intervention. Many IDI and FGD participants mentioned learning information about the disease at their workplace, through the VRA intervention, or through other members in the community who had gotten the disease.

*When we were working at Banana farm…they informed us that there are some snails in the river which cause this disease so if someone is frequently swimming in the river, they are also in the river – it is very easy for people to get this disease. (Community member, Asuogyaman)*

*I also know it’s from swimming in the river because children also get infected since they swim in the river anytime they go to fetch water. (FGD adult male, South Tongu)*

*I remember that sometime ago, experts from the VRA came to educate us on urinating blood. They explained that there are organisms in the river that can cause this condition. When our children, or even the elders, swim in it, bathe with it, or use the water without boiling or treating it, it can lead to this condition. (FGD adult female, Asuogyaman)*

While changes in knowledge of how the disease is acquired, misconceptions, and comprehensive knowledge did not change significantly after the VRA intervention, correct knowledge of how to prevent the disease significantly increased from 38.85% to 45.95%. Correct knowledge of signs and symptoms associated with the disease significantly decreased from 86.25% to 75.72%. Table 6 details changes in knowledge about schistosomiasis before and after the VRA intervention among survey respondents (Table 6).

**Table 6.** Knowledge about schistosomiasis before and after Volta River Authority (VRA) intervention

	Pre-intervention phase, *N* = 960		Post-intervention phase, *N* = 906		*p*
	Frequency	Percentage	Frequency	Percentage	
Have knowledge of how schistosomiasis is acquired					
Yes^a^	804	92.31	685	92.32	0.109
No	67	7.69	57	7.68	
Have correct knowledge of signs and symptoms associated with schistosomiasis					
Yes	828	86.25	686	75.72	0.000
No	132	13.75	220	24.28	
Have correct knowledge of schistosomiasis prevention					
Yes^a^	303	38.85	306	45.95	0.000
No	477	61.15	360	54.05	
Misconception about how schistosomiasis is acquired					
Yes	125	13.02	110	12.14	0.567
No	835	86.98	796	87.86	
Comprehensive knowledge of schistosomiasis					
Yes	294	30.62	290	32.01	0.519
No	666	69.38	616	67.99	

a Valid *n* differs due to missing data: Knowledge of how schistosomiasis is acquired: pre (= 871), post (= 742); correct knowledge of how to prevent schistosomiasis: pre (= 780), post (= 666).

Analysis of interviews with health workers revealed that knowledge of prevention does not necessarily align with community attitudes and practices regarding the disease. Based on the IDIs and FGDs, many community members perceived schistosomiasis as a normal, endemic condition. Community members with this attitude may not seek care immediately, leading to complications of the disease.

*There has been a lot of education about schistosomiasis and the mode of transmission so for now they know but they will tell you they don’t have any other source of water. (Health worker, Shai Osudoku)*

*They know that you get schisto when you wade through the water. You get schisto when you swim. But you see, yes, and so what? Nothing is happening to me, so it’s not a bother to me. (Health worker, Asuogyaman)*

*But for the schisto, we have some communities that the condition is endemic with them. The issue is that they hardly see it as a sickness. So we normally see them at the latter stage of the condition. That is when they started having other secondary infections. (Health worker, Asuogyaman)*

Healthcare worker confidence in discussing schistosomiasis after the MDA intervention training significantly increased from 86.78% to 97.19%. Those interviewed felt the training significantly enhanced their knowledge of the disease and expressed interest in future refresher sessions; 42.06% of healthcare workers who participated in the training strongly agreed and 46.73% agreed when asked whether their expectations of the training were met.

*Yes, because during the TOT which is the training of trainers that is how I got to know the long-term female disease and I was moved because it was something new to me so I will take part again. (Health worker, Shai Osudoku)*

*The thing is that when we went for the training, they taught us everything. But unfortunately, I forgot most of the things. (Health worker, South Tongu)*

*I wish they would do a revise. (Health worker, Shai Osoduku)*

### Assessing adoption of the MDA programme

**Figure 1.**
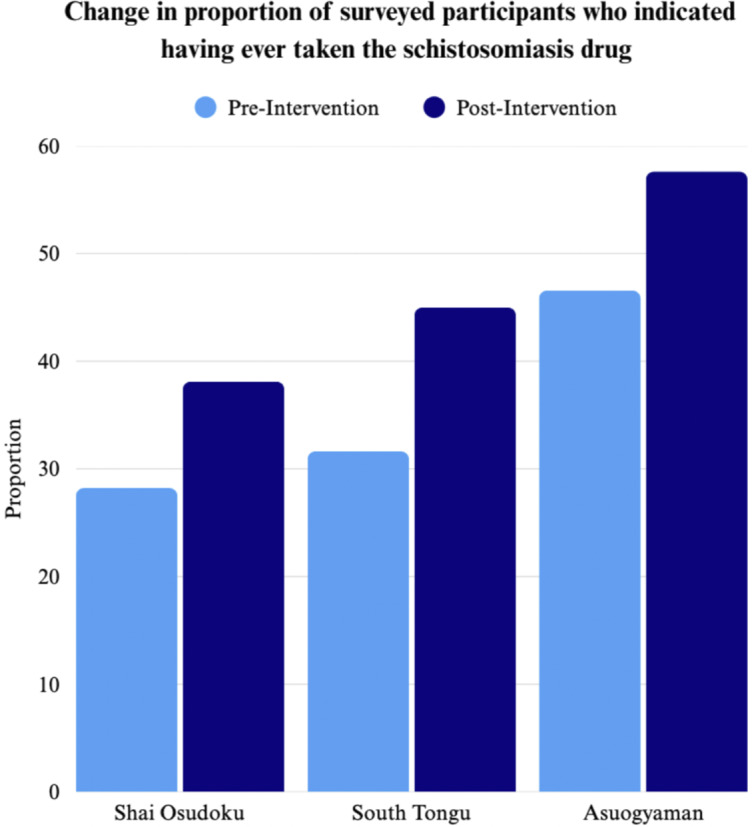
Change in proportion of surveyed participants who indicated having ever taken the schistosomiasis drug.

The proportion of surveyed community members who indicated having ever taken the schistosomiasis drug increased significantly across all three districts as displayed in Figure 1. Health workers noted that community members were more involved in this MDA compared to prior MDAs and worked to mobilize fellow community members, contributing to a wider acceptance of the intervention.

*They are happy and they mobilize the people because they know they have been battling with this disease and luckily enough drugs are free for that one. (Health worker, Shai Osduoku)*

*But then, some communities helped. They understood. The assembly members were also good. They were providing water for people to use to take their drug. They were also going around to call the people, going around from community to community, house to house, telling them that they should take their drugs. (Health worker, South Tongu)*

**Figure 2.**
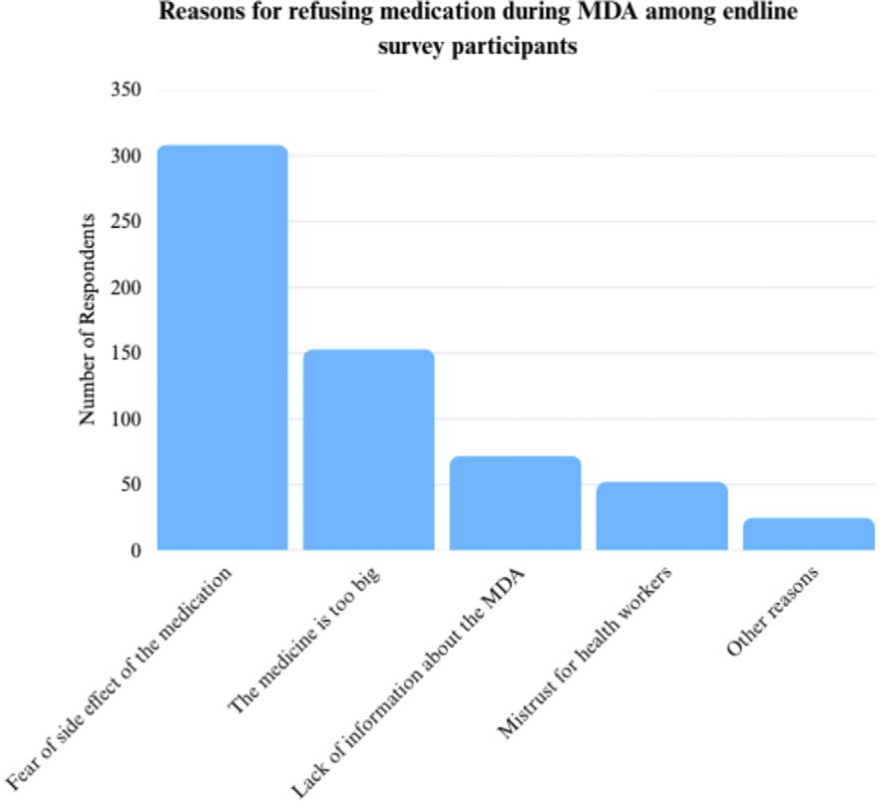
Reasons for refusing medication during MDA among endline survey participants.

Drug uptake remained a challenge, with 13.23% of community members expressing they did not want to take the medication, citing various reasons including fear of the side effects from taking the drug and the large size or bitter taste of the medicine as shown in Figure 2. A common challenge reported in IDIs and FGDs was the lack of food provided with the drug. Although distributors were trained to advise participants to eat a heavy meal beforehand to prevent side effects such as vomiting and dizziness, many community members struggled to prepare or afford one, particularly the school children.

*…Those administering the drugs should provide the children with heavy meals to eat. Some of the children don’t have much money, so they can only afford light foods. Taking a high-dose medication on an empty stomach or with light foods makes it harder for the children. (FGD, adult female, Asuogyaman)*

Another common challenge reported was drowsiness as a side effect of the drug. Many IDI and FGD participants, having experienced drowsiness after taking the praziquantel during prior MDAs, chose to refuse the drug this time around for fear of missing out on a day of work. This challenge was most felt when the drug distribution took place in the early morning. Several IDI and FGD participants suggested having drug distributions at different points in the day to minimize such challenges and to maximize community availability.

*Some of the drugs cause a lot of people to sleep. But there are some people who do not have that time for sleep because they have a lot of things to do. Like me, once I wake up, I clean around and do all sorts of work in the house. (FGD, adult female, Asuogyaman)*

*I think during the recent distributions that we did, most of the people were complaining about how the previous praziquantel distribution was done like the way it made them felt, like they felt sick and all that so of them were feeling reluctant to take the praziquantel. (Health worker, Asuogyaman)*

### Understanding implementation of the MDA programme

Community members mentioned receiving information about the MDA from community information centres (CIC), health worker home visits, community durbars, and school. Many participants indicated that they did not have access to television and radio. Information dissemination through churches and assembly leaders was cited as a successful strategy only if church leaders were consulted and educated on the importance of the drug.

*Sometimes, the nurses send letters to the churches, and the pastors read and explain the information in the letters. They tell the congregation that the river we swim in or drink from isn’t safe, so it’s important to boil the water before using it for drinking or bathing as well as the diseases that can be contracted if we disobey. Most pastors, including mine, read and explain the letters to us. (FGD adult female, Asuogyaman)*

*There were churches who were also against medication. So they don’t want their people to take the medication. So that was one of the challenges we actually faced. But when we spoke to some of the church leaders, they allowed it. So it depends on…that’s why the social mobilization is very important. (VRA Staff, Asuogyaman)*

Other successful community engagement strategies included involving community members by showing them urine samples under a microscope to visually demonstrate the health issue when testing.

*One of the days, we went with the VRA team. And then they even went there to take their urine sample to observe the X-rays. Sometimes they even let them look at it under the microscope. And when they saw that, yes, there is something in my urine, they would go out and even tell their colleagues that this thing is real. So the involvement was good this time around. And then I think it also helped in the coverages as well. This time, a lot of people took the medication, unlike the traditional ones we’ve been doing. (Health worker, Asuogyaman)*

### Assessing maintenance of the MDA programme

IDI and FGD participants identified several challenges and offered suggestions for improvement of the MDA and schistosomiasis control in the Volta Region. One of the challenges identified for the MDA was related to the drug distributors, which consisted of health workers, volunteers, and teachers for the school-based MDA. Several participants raised concerns about the role of teachers as distributors, noting that the teachers were untrained and unprepared to distribute the drug or that it was posing a challenge by taking away from their instructional time. Others said that health workers should be present during the school-based MDA in the event of side effects, which teachers felt unprepared to handle.

*There should be some more training. If they want the teachers to do it, then they must come down and then give us the necessary training. (Teacher, Shai Osudoku)*

*Because the time that we use to administer this drug is an instructional hour. And I’ll need that time to be inside the classroom and I’ll be there giving drugs. (SHEP Coordinator, Shai Osudoku)*

IDI and FGD participants also advocated for more community education, suggesting that the drug should be distributed more frequently and that the MDA should be accompanied by increased education well ahead of time. Moreover, participants suggested that parents should be a key target of community education due to their influence on their children’s behaviour. Other strategies to enhance community engagement included educational outreach by existing local groups, such as the mat weavers’ and fishermen’s associations. Participants also suggested that education on schistosomiasis could be done by someone who had the disease.

*The health workers need to educate the community about this disease and they need to inform us ahead about the distribution of the drug and they need to be distributing the drug frequently so that this disease can be prevented. They shouldn’t just give us the medicine once in a while and come back in a long time to distribute the drug again, they need to be frequently distributing the drug to us so that we can all prevent this disease. (FGD adult female, Asuogyaman)*

*We need to talk to the parents first on the day of distribution. They didn’t even come because the parents have this mentality that medications from the government are not good so it makes the kids not to come for medication. (Health worker, Shai Osudoku)*

*Get someone with the schisto, someone with the problem. Let the person do the education in this particular community. (Health worker, Shai Osudoku)*

Further suggestions for MDA improvement were related to the drug itself, with many participants suggesting that food and drink should be provided alongside the drug to minimize side effects and combat the bitter taste and unpleasant scent of the praziquantel.

Interviewed health workers suggested the health worker training component of the intervention could be improved by training health workers in batches so that more people could attend, having refresher training sessions, providing more training on female genital schistosomiasis, and ensuring that the training site is accessible and close to the community.

The main challenge identified for schistosomiasis control was the issue of the root cause of the disease: infected water bodies. IDI and FGD participants noted that although people are aware of the disease and of the effectiveness of the MDA in reducing schistosomiasis prevalence, they would still return to the river and get re-infected because it is their only source of water.

*And the drug too, it’s effective because most often when they take the medication and go back to the community, you realize that their prevalence has come down. Just that the source of infection is still there. So they go back and get infected again. (Health worker, Asuogyaman)*

*[Schistosomiasis] can be cured but the challenge is that, since we keep entering the river for fishing and we use the water frequently, it is difficult to eradicate. Unless the river is treated, more pipe water is provided, and those companies redirect their drainage from the river, we can’t eradicate the sickness totally. (FGD adult male, Asuogyaman)*

The main suggestions for this challenge were related to WASH measures, specifically the elimination of parasites from the water, provision of potable water, and sanitary infrastructure, and education not just on the drug but on the importance of sanitation and hygiene.

## Discussion

This study aimed to evaluate the impact of an evidence-based context-specific MDA-related intervention for schistosomiasis control in three districts of Ghana using the RE-AIM framework. The findings revealed a large decrease in schistosomiasis prevalence in all three districts, a significant increase in knowledge about prevention measures but a significant decrease in correct knowledge of signs and symptoms, high health worker satisfaction with the training, and positive community reception of the MDA.

High levels of training among both district health management staff and CDDs were attained, accompanied by high reported satisfaction with the training by health workers and increased health worker knowledge. These findings suggest that Domain 3 of the VRA intervention, which pertains to the training component, was implemented effectively (see Table 1 for Domain details).

The intervention did not appear to significantly affect community knowledge regarding disease acquisition, misconceptions of the disease, or comprehensive knowledge on the disease. Notably, the proportion of respondents with correct knowledge of signs and symptoms of schistosomiasis significantly decreased, which may indicate that community educators prioritized other knowledge areas during this intervention, or that the intervention was not directly correlated with knowledge levels in the community. However, overall knowledge in each of these areas among the community surveyed was already relatively high prior to the intervention. This raises the possibility that, if the intervention influenced knowledge at all, its impact may have been concentrated on addressing knowledge gaps, particularly in relation to disease prevention, where baseline knowledge was lower compared to knowledge of disease acquisition and symptoms. While definitive conclusions about the effects of the VRA intervention on community knowledge cannot be drawn from the quantitative data, themes from the IDIs and FGDs suggested that community education could serve as a tool to shift attitudes towards schistosomiasis by de-normalizing the disease and prompting earlier care-seeking behaviour. IDI and FGD respondents also indicated that community involvement in the MDA increased during the VRA intervention, aligning with the goal of Domain 1. Many respondents reported receiving MDA-related information through the communication channels outlined in Domain 2, which could suggest that the community engagement and social mobilization components of the VRA intervention strategies were implemented effectively (see Table 1 for Domain details).

VRA staff largely emphasized the benefits of partnering with the Ghana Health Service for the MDA, noting that the collaboration leveraged shared resources to expand coverage and reach a common goal. While coverage largely improved (though coverage data remain a noted limitation: see Limitations) barriers to drug uptake persisted. Although many respondents viewed the lack of cost associated with the drug as a facilitator, IDIs and FGDs revealed several apparent opportunity costs associated with taking the drug – such as time away from work or challenges securing food beforehand – that some community members could not afford to take. IDI and FGD respondents who understood the importance of the drug, were aware of the MDA, had access to food to take with the drug, or could afford to take a day off from work or school indicated they were more willing to take the drug, suggesting that these opportunity costs may influence an individual’s decision to partake in the MDA. This suggests that Domain 4 of the VRA intervention, which focuses on the drug distribution process, could be revised to take into account these apparent opportunity costs to drug uptake (see Table 1 for Domain details). Moreover, coverage decreased in South Tongu, which district health authorities attributed to drug shortages. At the same time, prevalence also decreased, which may reflect delayed effects of previous higher coverage MDAs or other contextual factors influencing transmission.

The challenge of intense side effects, bitterness, and large size of the drug praziquantel has been noted in prior studies on schistosomiasis MDA [14]. However, in February 2025, the Paediatric Praziquantel Consortium developed a new formula for praziquantel that decreases the bitterness of the drug and makes it more suitable for distribution to children [15]. Based on community suggestions in the IDIs and FGDs, procuring this version of praziquantel, providing heavy meals during the MDA to both minimize side effects and serve as an economic incentive, and using churches not only to disseminate information but to disseminate the drug may combat the challenges identified by participants in this study and increase praziquantel uptake.

The findings of this study are broadly consistent with existing research in Ghana on the implementation and impact of MDA-related intervention for schistosomiasis. For example, a study conducted in communities along the Volta River and in Asuogyaman District reported through IDIs and FGDs that participants expressed willingness to participate in the MDA yet held concerns about praziquantel uptake due to past experiences with adverse side effects [16]. Similarly, a recent impact assessment of schistosomiasis MDA in Ghana, based on three national surveys conducted between 2007 and 2024, found that schistosomiasis prevalence decreased substantially from baseline to first impact assessment and remained low through the second impact assessment, which the study attributed to the introduction of MDA in Ghana in 2010 [17]. However, this RE-AIM study suggests that knowledge of schistosomiasis may not necessarily translate into preventive behaviour, which differs from findings of an assessment of risk factors for schistosomiasis among school-aged children in Pru East, Ghana [18]. Although survey respondents in this study demonstrated relatively high levels of knowledge of schistosomiasis acquisition both before and after the VRA intervention, several IDI and FGD respondents suggested that this knowledge does not necessarily drive community behaviours, which they described as more strongly shaped by structural constraints. Interviewed community members spoke about continuing to use the river despite understanding the risk of re-infection because the river is essential to their daily lives and economic livelihood. IDI and FGD respondents suggested that complementary interventions beyond MDA and MDA education are needed, particularly those that address the root cause of the disease. Although the VRA designed a WASH intervention using a similar approach to the MDA – based on needs assessments and intervention mapping – participants of this study reported a preference for the river even when provided with potable water. As a result, the availability of pipe water or boreholes may be insufficient to change behaviours rooted in necessity and tradition. Future interventions should therefore consider incorporating vector control measures, such as the removal of the water weeds that harbour the snail hosts, the provision of sanitary infrastructure, and targeted education on hygiene practices. The VRA should also consider strengthening their current alternative income schemes to both protect the water body and decrease economic dependence on the river.

### Strengths

This study has demonstrated the importance and utility of the RE-AIM framework as a tool for public health intervention evaluation. It is the first study to use the RE-AIM framework to evaluate a schistosomiasis control programme. The use of both quantitative and qualitative data in this study strengthened its findings by allowing the discovery of themes relevant to the pillars of the framework. Survey data were enhanced and contextualized by the IDIs and FGDs in a way that added nuance to themes such as barriers to drug uptake, community engagement, and perceptions of schistosomiasis.

### Limitations

As with most studies examining behaviours and attitudes, the findings of this study could be influenced by social desirability bias, whereby participants of the survey, IDIs, or FGDs may have omitted or minimized behaviours or attitudes that they thought were unfavourable. While the IDIs and FGDs offered valuable nuance and detail on individual-level beliefs and attitudes, these methods did not permit extrapolation to the broader population. Additionally, MDA coverage data were obtained directly from individual districts rather than from a standardized database. As a result, the data for pre-intervention MDA coverage vary considerably – some districts reported only school-based MDA coverage, others provided data from MDAs conducted over 5 years ago, and several data points may have been incomplete or missing. This lack of standardization limits the ability to draw reliable conclusions about changes in MDA coverage from pre- to post-intervention. Finally, the time between baseline data collection and endline data collection was 1 year, which limited the assessment of intervention maintenance and long-term sustainability.

## Conclusion

The findings of this study on the reach, effectiveness, adoption, implementation, and maintenance of the context-specific evidence-based VRA intervention suggest that Domains 1, 2, and 3 – related to community engagement, social mobilization, and MDA-related training, respectively – were implemented effectively, whereas Domain 4, pertaining to the drug distribution process, could be improved upon. The findings also suggest that the intervention contributed to decreases in schistosomiasis prevalence and may have helped address community knowledge gaps about the disease as well as supported increased community awareness and acceptance of the MDA. While challenges to drug uptake remained, participants pointed to the broader challenge of addressing the underlying source of schistosomiasis: infected bodies of water. These findings suggest that future research and interventions could explore strategies such as reduction of parasites from the Volta River, decreasing community economic and domestic reliance on the river, and ensuring hygienic practices in schistosomiasis endemic areas.

## Data Availability

The data that support the findings of this study are available from the authors upon reasonable request.
